# Supplementary material for: Amorphous topological superconductivity in a Shiba glass
Source: Nat Commun. 2018 May 29;9:2103. doi: 10.1038/s41467-018-04532-x (PMC5974401; doi:10.1038/s41467-018-04532-x)
Supplement: Supplementary file 1 — Supplementary Information [file 41467_2018_4532_MOESM1_ESM.pdf]

Supplementary Figures

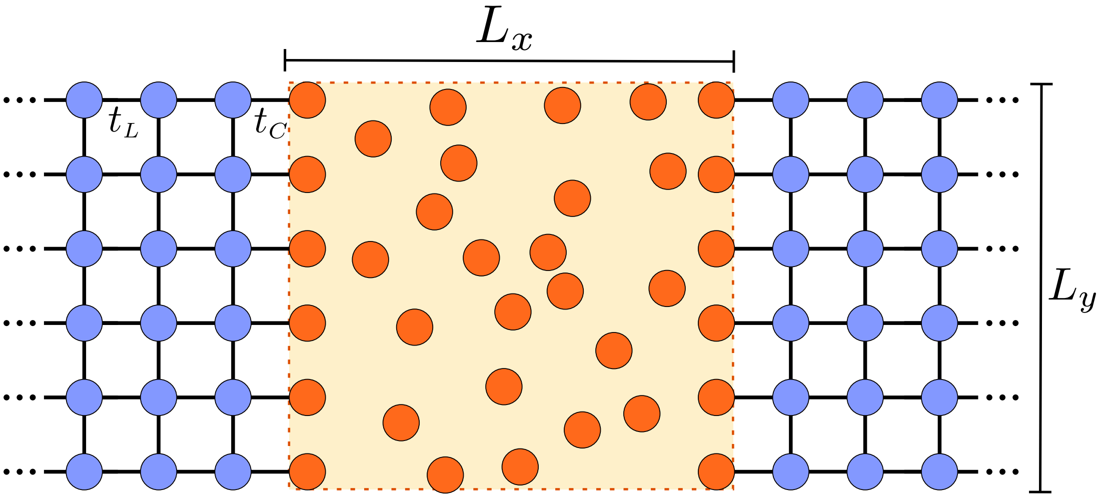

Supplementary Figure 1: Schematic figure of the conductance calculation setup. The box outlined in orange denotes the scattering area, with long-range hoppings, whereas the leads feature only simple nearest-neighbour hopping. As seen in the figure, the border sites of the scattering area are fixed, and are the only sites coupled to the leads.

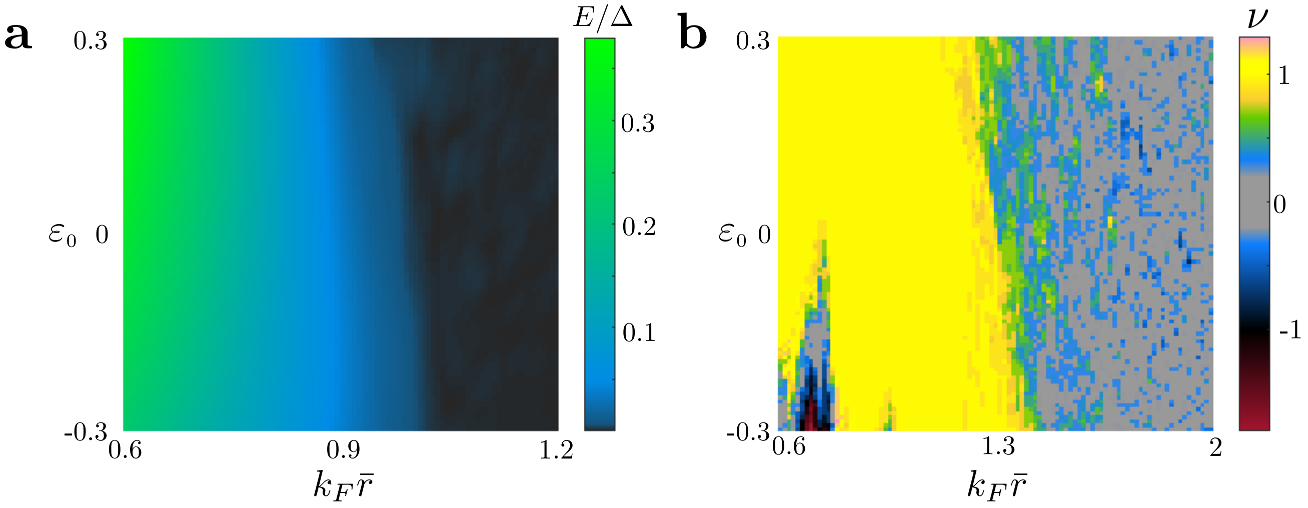

Supplementary Figure 2: Topological phase diagrams at different parameters. **a,** Energy gap diagram for a finite system. In the topological phase, we observe a finite energy gap, while the trivial phase is on average gapless. The adatom number is held fixed at 600, with $k_{\mathrm{F}}\xi = \frac{4\pi}{5}$ and $\lambda = 0.2$. The displayed diagram is an average over 10 configurations. **b,** Chern number diagram for a Shiba glass with $k_{\mathrm{F}}\xi = \frac{12}{5}\pi$ and $\lambda = 0.1$. Other parameters are the same as in Supplementary Fig. 2a.

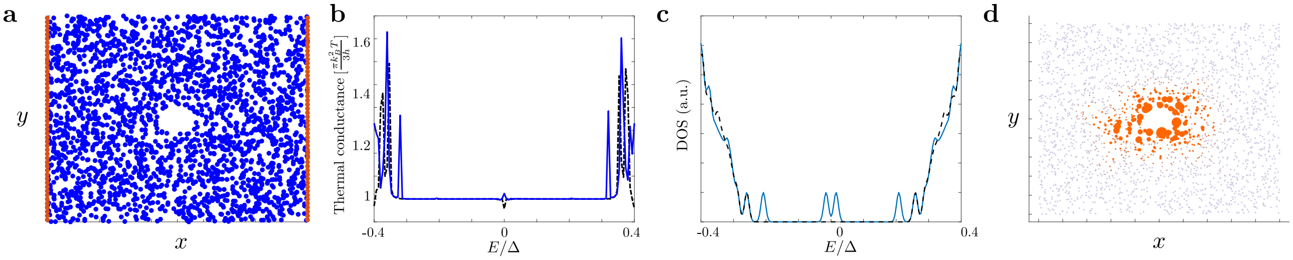

Supplementary Figure 3: Realisation of a gapless system with nonzero mobility gap. **a,** A map of the lattice site configuration, with a small antipuddle in the middle. The sites colored in red are connected to semi-infinite leads. **b,** Heat conductance as a function of energy. From the figure, it can be observed that the heat conductance is quantized for energies $|E| \lesssim 0.3\Delta$. This quantized conductance plateau is only present in the topological phase. As a comparison, the dashed line corresponds to the conductance with the hole filled. As can be seen, the presence of the hole does not significantly impact the quantization. **c,** Density of states (DOS) for a system consisting of the disordered part of the scattering region, the blue sites in **a**, with periodic boundary conditions. The low energy states correspond to an edge mode around the antipuddle in the center. Periodic boundary conditions have been implemented to remove the effect of the heat conducting edge modes at the edges of the strip. From the figure it is clear that the system is gapless or nearly gapless, with DOS peaks at low energies. The dashed line corresponds to the DOS obtained for the same system with the hole filled; in this case, the low-energy peaks corresponding to antipuddle bound states are not present. **d,** Local density of states for the system described in **c**, i.e., the scattering region of **a** with periodic boundary conditions, integrated over energies $[-0.1\Delta, 0.1\Delta]$. Observe that the low-energy states are bound at the antipuddle in the middle, and hence do not significantly disrupt the overall quantized heat conductance.

Supplementary Note 1: Microscopic derivation of the model

In this section, we present a microscopic derivation of the effective Shiba glass Hamiltonian studied in the main text. The derivation follows the spirit of Supplementary Ref. [1], but proceeds differently since we are considering 2D systems with a spin-orbit coupling and arbitrary spin textures rather than 1D helical magnetic chains. Similar 1D [2] and 2D [3, 4] systems have been previously studied on regular lattices with ferromagnetic textures where significant simplification occurs. Our starting point is the mean-field Bogoliubov-de Gennes equation for magnetic impurities on a 2D s-wave superconductor with Rashba spin-orbit coupling,

$$\left[\xi_k\tau_z + \alpha_{\rm R}(\boldsymbol{\sigma}\times\mathbf{k})_z\tau_z + \Delta\tau_x + JS\sum_j(\hat{\mathbf{S}}_j\cdot\boldsymbol{\sigma})\delta(\mathbf{r}-\mathbf{r}_j)\right]\Psi = E\Psi \tag{1}$$

where $\xi_k = \frac{k^2}{2m} - \mu$ represents the kinetic energy and chemical potential, $\alpha_{\rm R}$ the spin-orbit coupling, Δ the superconducting pairing amplitude, and J and S are the exchange coupling and the magnitude of the classical impurity spin, respectively. The arbitrary positions of N magnetic moments are labelled by $\mathbf{r}_j$ and they point in the direction defined by the unit vectors $\hat{S}_j$. The τ and σ matrices correspond to Pauli matrices in particle-hole and spin subspaces; the vector basis is $\Psi = (\psi_\uparrow, \psi_\downarrow, \psi_\downarrow^\dagger, -\psi_\uparrow^\dagger)^T$. By Fourier transforming, the equation can be re-expressed as

$$\Psi(\mathbf{r}_i) = JS\sum_j\int\frac{d\mathbf{k}}{(2\pi)^2}\mathrm{e}^{\mathrm{i}\mathbf{k}\cdot\mathbf{r}_{ij}}G_0(\mathbf{k})(\hat{\mathbf{S}}_{\mathbf{j}}\cdot\sigma)\Psi(\mathbf{r}_j), \tag{2}$$

where $\mathbf{r}_{ij} = \mathbf{r}_i - \mathbf{r}_j$, and $G_0(\mathbf{k})$ is the Green's function of the superconductor without impurities. Separating the terms dependent on $\mathbf{r}_i$, the above can be written as

$$\left[\hat{\mathbf{S}}_i\cdot\boldsymbol{\sigma} - \alpha\frac{E+\Delta\tau_x}{\sqrt{\Delta^2-E^2}}\right]\Psi(\mathbf{r}_i) = -\sum_{j\neq i}(\hat{\mathbf{S}}_i\cdot\boldsymbol{\sigma})J_E(\mathbf{r}_{ij})(\hat{\mathbf{S}}_j\cdot\boldsymbol{\sigma})\Psi(\mathbf{r}_j), \tag{3}$$

where $\alpha = \frac{1}{2}JSm$ and

$$J_E(\mathbf{r}) = \frac{JS}{2}\int\frac{d\mathbf{k}}{(2\pi)^2}\mathrm{e}^{\mathrm{i}\mathbf{k}\cdot\mathbf{r}}\left[G_0^+(\mathbf{k}) + G_0^-(\mathbf{k})\right]. \tag{4}$$

Due to the Rashba coupling, the substrate Green's function splits into two helical components

$$G_0^\nu = \frac{E + \xi_\nu \tau_z + \Delta\tau_x}{E^2 - \xi_\nu^2 - \Delta^2}\left[1 + \nu(\frac{k_y}{k}\sigma_x - \frac{k_x}{k}\sigma_y)\right], \tag{5}$$

where the helical branches $\nu = \pm 1$ have dispersions $\xi_\nu = \xi_k + \nu\alpha_{\rm R} k$. Evaluation of the Green's function can be carried out by linearizing at the Fermi surface $\xi_\nu = 0$ which yields

$$J_E(\mathbf{r}) \approx \frac{1}{2} JSm \sum_{\nu=\pm 1}(1 - \nu\frac{\lambda}{\sqrt{1+\lambda^2}})\int\frac{d\xi}{2\pi}\int\frac{d\theta}{2\pi}\mathrm{e}^{\mathrm{i}k_\nu r\cos(\varphi-\theta)}\frac{E + \xi_\nu\tau_z + \Delta\tau_x}{E^2 - \xi_\nu^2 - \Delta^2}\left[1 + \nu(\sin\theta\sigma_x - \cos\theta\sigma_y)\right], \tag{6}$$

where φ is the angle between $\mathbf{r}$ and the x axis. Hence, we can write

$$\begin{aligned} J_E(\mathbf{r}_{ij}) = \frac{\alpha}{2}\Big[&(E + \Delta\tau_x)(I_1^+(r_{ij}) + I_1^-(r_{ij})) - \mathrm{i}\tau_z(\frac{y_i - y_j}{r_{ij}}\sigma_x - \frac{x_i - x_j}{r_{ij}}\sigma_y)(I_0^+(r_{ij}) - I_0^-(r_{ij})) \\ &+ \tau_z(I_2^+(r_{ij}) + I_2^-(r_{ij})) + (E + \Delta\tau_x)(\frac{y_i - y_j}{r_{ij}}\sigma_x - \frac{x_i - x_j}{r_{ij}}\sigma_y)(I_3^+(r_{ij}) - I_3^-(r_{ij}))\Big], \end{aligned} \tag{7}$$

where we have defined the integrals

$$\begin{aligned} I_0^\pm(r) &= \mathrm{i}\frac{N_\pm}{2\pi^2}\int_{-\infty}^{\infty} d\xi\int_0^{2\pi} d\varphi\frac{\xi\mathrm{e}^{\mathrm{i}k^\pm r\cos\varphi + \mathrm{i}\varphi}}{E^2 - \xi^2 - \Delta^2} = N_\pm\,\mathrm{Re}\left[\mathrm{i}J_1(k_{\rm F}^\pm r + \mathrm{i}\frac{r}{\xi_E}) + \frac{2}{\pi} - H_1(k_{\rm F}^\pm r + \mathrm{i}\frac{r}{\xi_E})\right] \\ I_1^\pm(r) &= \frac{N_\pm}{2\pi^2}\int_{-\infty}^{\infty} d\xi\int_0^{2\pi} d\varphi\frac{\mathrm{e}^{\mathrm{i}k^\pm(\xi)r\cos\varphi}}{E^2 - \xi^2 - \Delta^2} = \frac{-N_\pm}{\sqrt{\Delta^2 - E^2}}\,\mathrm{Re}\left[J_0(k_{\rm F}^\pm r + \mathrm{i}\frac{r}{\xi_E}) + \mathrm{i}H_0(k_{\rm F}^\pm r + \mathrm{i}\frac{r}{\xi_E})\right] \\ I_2^\pm(r) &= \frac{N_\pm}{2\pi^2}\int_{-\infty}^{\infty} d\xi\int_0^{2\pi} d\varphi\frac{\xi\mathrm{e}^{\mathrm{i}k^\pm(\xi)r\cos\varphi}}{E^2 - \xi^2 - \Delta^2} = N_\pm\,\mathrm{Im}\left[J_0(k_{\rm F}^\pm r + \mathrm{i}\frac{r}{\xi_E}) + \mathrm{i}H_0(k_{\rm F}^\pm r + \mathrm{i}\frac{r}{\xi_E})\right] \\ I_3^\pm(r) &= \frac{N_\pm}{2\pi^2}\int_{-\infty}^{\infty} d\xi\int_0^{2\pi} d\varphi\frac{\mathrm{e}^{\mathrm{i}k^\pm(\xi)r\cos\varphi + \mathrm{i}\varphi}}{E^2 - \xi^2 - \Delta^2} = -\mathrm{i}\frac{N_\pm}{\sqrt{\Delta^2 - E^2}}\,\mathrm{Im}\left[\mathrm{i}J_1(k_{\rm F}^\pm r + \mathrm{i}\frac{r}{\xi_E}) + \frac{2}{\pi} - H_1(k_{\rm F}^\pm r + \mathrm{i}\frac{r}{\xi_E})\right]. \end{aligned}$$

In the above, we have used the dimensionless spin-orbit coupling $\lambda = \frac{\alpha_{\rm R}}{v_{\rm F}}$ and the superconducting coherence length $\xi_E = \frac{v_{\rm F}}{\sqrt{\Delta^2 - E^2}}$ as well as the densities of states of the two helical branches $N_\pm = 1 \mp \frac{\lambda}{\sqrt{1+\lambda^2}}$, their linearised dispersions $k^\pm(\xi) = \frac{\xi}{v_{\rm F}} + k_{\rm F}^\pm$, as well as their Fermi wave vectors $k_{\rm F}^\pm = k_{\rm F}(\sqrt{1+\lambda^2} \mp \lambda)$. In general, the integrals above decay with site distance as $f(r) \propto \mathrm{e}^{-r/\xi_E}/\sqrt{r}$; however, I_0 and I_2 contain terms decaying asymptotically as r^{-2} and r^{-1}, respectively.

We now introduce the quantities

$$S_{ij} = \sum_\nu N_\nu\left[J_0(k_{\rm F}^\nu r_{ij} + \mathrm{i}\frac{r_{ij}}{\xi_E}) + \mathrm{i}H_0(k_{\rm F}^\nu r_{ij} + \mathrm{i}\frac{r_{ij}}{\xi_E})\right] \tag{8}$$

$$A_{ij} = \sum_\nu \nu N_\nu\left[\mathrm{i}J_1(k_{\rm F}^\pm r_{ij} + \mathrm{i}\frac{r_{ij}}{\xi_E}) + \frac{2}{\pi} - H_1(k_{\rm F}^\pm r_{ij} + \mathrm{i}\frac{r_{ij}}{\xi_E})\right] \tag{9}$$

in terms of which the integrals can be written

$$\mathrm{Re}(S_{ij}) = -\sqrt{\Delta^2 - E^2}(I_1^+(\mathbf{r}_{ij}) + I_1^-(\mathbf{r}_{ij})) \tag{10}$$

$$\mathrm{Im}(S_{ij}) = I_2^+(\mathbf{r}_{ij}) + I_2^-(\mathbf{r}_{ij}) \tag{11}$$

$$\mathrm{Re}(A_{ij}) = I_0^+(\mathbf{r}_{ij}) - I_0^-(\mathbf{r}_{ij}) \tag{12}$$

$$\mathrm{i}\,\mathrm{Im}(A_{ij}) = -\sqrt{\Delta^2 - E^2}(I_3^+(\mathbf{r}_{ij}) - I_3^-(\mathbf{r}_{ij})). \tag{13}$$

To simplify notation, we define the operator $\kappa_{ij} = \frac{y_i - y_j}{r_{ij}}\sigma_x - \frac{x_i - x_j}{r_{ij}}\sigma_y$. Supplementary Eq. (3) now takes the form

$$\begin{aligned} \left[\hat{\mathbf{S}}_i\cdot\boldsymbol{\sigma} - \alpha\frac{E + \Delta\tau_x}{\sqrt{\Delta^2 - E^2}}\right]\Psi(\mathbf{r}_i) = -\frac{\alpha}{2}\sum_{j\neq i}(\hat{\mathbf{S}}_i\cdot\boldsymbol{\sigma})\Big[&-\frac{E + \Delta\tau_x}{\sqrt{\Delta^2 - E^2}}\,\mathrm{Re}\,S_{ij} - \mathrm{i}\tau_z\kappa_{ij}\,\mathrm{Re}\,A_{ij} \\ &+ \tau_z\,\mathrm{Im}\,S_{ij} - \mathrm{i}\frac{E + \Delta\tau_x}{\Delta^2 - E^2}\kappa_{ij}\,\mathrm{Im}\,A_{ij}\Big](\hat{\mathbf{S}}_j\cdot\boldsymbol{\sigma})\Psi(\mathbf{r}_j). \end{aligned} \tag{14}$$

The next step is transforming Supplementary Eq. (3) to the local BdG basis $\{|+\rangle_{\tau_x}\otimes|\uparrow_j\rangle, |-\rangle_{\tau_x}\otimes|\downarrow_j\rangle, |+\rangle_{\tau_x}\otimes|\downarrow_j\rangle, |-\rangle_{\tau_x}\otimes|\uparrow_j\rangle\}$. The spin states are defined in terms of the spin directions $\hat{\mathbf{S}}_j = (\sin\theta_j\cos\varphi_j, \sin\theta_j\sin\varphi_j, \cos\theta_j)$ as

$$|\uparrow_j\rangle = \frac{1}{\sqrt{N}}\begin{pmatrix} \mathrm{e}^{-\mathrm{i}\frac{\varphi_j}{2}}\cos\frac{\theta_j}{2} \\ \mathrm{e}^{\mathrm{i}\frac{\varphi_j}{2}}\sin\frac{\theta_j}{2} \end{pmatrix} \qquad |\downarrow_j\rangle = \frac{1}{\sqrt{N}}\begin{pmatrix} -\mathrm{e}^{-\mathrm{i}\frac{\varphi_j}{2}}\sin\frac{\theta_j}{2} \\ \mathrm{e}^{\mathrm{i}\frac{\varphi_j}{2}}\cos\frac{\theta_j}{2}. \end{pmatrix}, \tag{15}$$

and their matrix elements are

$$\begin{aligned}
\langle\uparrow_i|\uparrow_j\rangle &= \cos\frac{\theta_i}{2}\cos\frac{\theta_j}{2}\mathrm{e}^{\mathrm{i}\frac{\varphi_i-\varphi_j}{2}} + \sin\frac{\theta_i}{2}\sin\frac{\theta_j}{2}\mathrm{e}^{-\mathrm{i}\frac{\varphi_i-\varphi_j}{2}} \\
\langle\uparrow_i|\downarrow_j\rangle &= -\cos\frac{\theta_i}{2}\sin\frac{\theta_j}{2}\mathrm{e}^{\mathrm{i}\frac{\varphi_i-\varphi_j}{2}} + \sin\frac{\theta_i}{2}\cos\frac{\theta_j}{2}\mathrm{e}^{-\mathrm{i}\frac{\varphi_i-\varphi_j}{2}} \\
\langle\uparrow_i|\sigma_x|\uparrow_j\rangle &= \cos\frac{\theta_i}{2}\sin\frac{\theta_j}{2}\mathrm{e}^{\mathrm{i}\frac{\varphi_i+\varphi_j}{2}} + \sin\frac{\theta_i}{2}\cos\frac{\theta_j}{2}\mathrm{e}^{-\mathrm{i}\frac{\varphi_i+\varphi_j}{2}} \\
\langle\uparrow_i|\sigma_y|\uparrow_j\rangle &= -\mathrm{i}\cos\frac{\theta_i}{2}\sin\frac{\theta_j}{2}\mathrm{e}^{\mathrm{i}\frac{\varphi_i+\varphi_j}{2}} + \mathrm{i}\sin\frac{\theta_i}{2}\cos\frac{\theta_j}{2}\mathrm{e}^{-\mathrm{i}\frac{\varphi_i+\varphi_j}{2}} \\
\langle\uparrow_i|\sigma_x|\downarrow_j\rangle &= \cos\frac{\theta_i}{2}\cos\frac{\theta_j}{2}\mathrm{e}^{\mathrm{i}\frac{\varphi_i+\varphi_j}{2}} - \sin\frac{\theta_i}{2}\sin\frac{\theta_j}{2}\mathrm{e}^{-\mathrm{i}\frac{\varphi_i+\varphi_j}{2}} \\
\langle\uparrow_i|\sigma_y|\downarrow_j\rangle &= -\mathrm{i}\cos\frac{\theta_i}{2}\cos\frac{\theta_j}{2}\mathrm{e}^{\mathrm{i}\frac{\varphi_i+\varphi_j}{2}} - \mathrm{i}\sin\frac{\theta_i}{2}\sin\frac{\theta_j}{2}\mathrm{e}^{-\mathrm{i}\frac{\varphi_i+\varphi_j}{2}}.
\end{aligned} \tag{16}$$

For notational convenience, we introduce the matrices

$$a_{ij}^{\sigma\sigma'} = \frac{1}{2}\left\langle\sigma_i|\sigma_j'\right\rangle \mathrm{Re}\, S_{ij} \tag{17}$$

$$b_{ij}^{\sigma\sigma'} = \frac{1}{2}\langle\sigma_i|\hat{\kappa}_{ij}|\sigma_j'\rangle \,\mathrm{Re}\, A_{ij} \tag{18}$$

$$c_{ij}^{\sigma\sigma'} = \frac{1}{2}\langle\sigma_i|\hat{\kappa}_{ij}|\sigma_j'\rangle \,\mathrm{Im}\, A_{ij} \tag{19}$$

$$d_{ij}^{\sigma\sigma'} = \frac{1}{2}\left\langle\sigma_i|\sigma_j'\right\rangle \mathrm{Im}\, S_{ij}, \tag{20}$$

where $|\sigma_j\rangle$ can be either $|\uparrow_j\rangle$ or$|\downarrow_j\rangle$. Now we can write Supplementary Eq. (14) in the $4N\times 4N$ form, defining $u \equiv (\Delta+E)/\sqrt{\Delta^2-E^2}$:

$$\begin{aligned}
&\begin{pmatrix} 1-\alpha u & 0 & 0 & 0 \\ 0 & -1+\alpha u^{-1} & 0 & 0 \\ 0 & 0 & -1-\alpha u & 0 \\ 0 & 0 & 0 & 1+\alpha u^{-1} \end{pmatrix}\Psi = -\alpha\left[\begin{pmatrix} -ua^{\uparrow\uparrow} & 0 & -ua^{\uparrow\downarrow} & 0 \\ 0 & u^{-1}a^{\downarrow\downarrow} & 0 & u^{-1}a^{\downarrow\uparrow} \\ -ua^{\downarrow\uparrow} & 0 & -ua^{\downarrow\downarrow} & 0 \\ 0 & u^{-1}a^{\uparrow\downarrow} & 0 & u^{-1}a^{\uparrow\uparrow} \end{pmatrix}\right. \\
&\qquad + \mathrm{i}\begin{pmatrix} 0 & -b^{\uparrow\downarrow} & 0 & -b^{\uparrow\uparrow} \\ -b^{\downarrow\uparrow} & 0 & -b^{\downarrow\downarrow} & 0 \\ 0 & -b^{\downarrow\downarrow} & 0 & -b^{\downarrow\uparrow} \\ -b^{\uparrow\uparrow} & 0 & -b^{\uparrow\downarrow} & 0 \end{pmatrix} + \begin{pmatrix} 0 & d^{\uparrow\downarrow} & 0 & d^{\uparrow\uparrow} \\ d^{\downarrow\uparrow} & 0 & d^{\downarrow\downarrow} & 0 \\ 0 & d^{\downarrow\downarrow} & 0 & d^{\downarrow\uparrow} \\ d^{\uparrow\uparrow} & 0 & d^{\uparrow\downarrow} & 0 \end{pmatrix} \\
&\qquad \left. + \mathrm{i}\begin{pmatrix} -uc^{\uparrow\uparrow} & 0 & -uc^{\uparrow\downarrow} & 0 \\ 0 & u^{-1}c^{\downarrow\downarrow} & 0 & u^{-1}c^{\downarrow\uparrow} \\ -uc^{\downarrow\uparrow} & 0 & -uc^{\downarrow\downarrow} & 0 \\ 0 & u^{-1}c^{\uparrow\downarrow} & 0 & u^{-1}c^{\uparrow\uparrow} \end{pmatrix}\right]\Psi.
\end{aligned} \tag{21}$$

Up to this point, the only approximation has been the standard evaluation of Green's functions by linearizing the dispersion at the Fermi level. Now we concentrate on the low energy behaviour in the vicinity of the gap center $|E| \ll \Delta$ and work in the linear order in E/Δ. We now project the problem to the basis of subgap Shiba states,[1, 2, 3, 4] which

corresponds to the upper-left $2N \times 2N$ block:

$$\begin{pmatrix} 1-\alpha u & 0 \\ 0 & -1+\alpha u^{-1} \end{pmatrix} \Psi = \alpha \Bigg[\begin{pmatrix} u a^{\uparrow\uparrow} & 0 \\ 0 & -u^{-1} a^{\downarrow\downarrow} \end{pmatrix} + \begin{pmatrix} \mathrm{i} u c^{\uparrow\uparrow} & 0 \\ 0 & -\mathrm{i} u^{-1} c^{\downarrow\downarrow} \end{pmatrix} + \begin{pmatrix} 0 & \mathrm{i} b^{\uparrow\downarrow} \\ \mathrm{i} b^{\downarrow\uparrow} & 0 \end{pmatrix} + \begin{pmatrix} 0 & -d^{\uparrow\downarrow} \\ -d^{\downarrow\uparrow} & 0 \end{pmatrix} \Bigg] \Psi. \tag{22}$$

In addition, we assume that the YSR states with energies $\varepsilon_0 = \frac{1-\alpha^2}{1+\alpha^2}\Delta$ determined by the dimensionless Shiba coupling α lie close to the gap center, so that $|1-\alpha| \ll 1$. Neglecting terms quadratic in these, we get

$$\begin{pmatrix} 1-\alpha-\frac{E}{\Delta} & 0 \\ 0 & -1+\alpha-\frac{E}{\Delta} \end{pmatrix} \Psi = \Bigg[\frac{E}{\Delta} \begin{pmatrix} a^{\uparrow\uparrow} + \mathrm{i}c^{\uparrow\uparrow} & 0 \\ 0 & a^{\downarrow\downarrow} + \mathrm{i}c^{\downarrow\downarrow} \end{pmatrix} + \alpha \begin{pmatrix} a^{\uparrow\uparrow} + \mathrm{i}c^{\uparrow\uparrow} & 0 \\ 0 & -a^{\downarrow\downarrow} + -\mathrm{i}c^{\downarrow\downarrow} \end{pmatrix} + \alpha \begin{pmatrix} 0 & -d^{\uparrow\downarrow} + \mathrm{i}b^{\uparrow\downarrow} \\ -d^{\downarrow\uparrow} + \mathrm{i}b^{\downarrow\uparrow} & 0 \end{pmatrix} \Bigg] \Psi, \tag{23}$$

where the submatrices a, b, c, d are to be evaluated at $E = 0$. We isolate the energy:

$$\frac{E}{\Delta} \begin{pmatrix} 1 + a^{\uparrow\uparrow} + \mathrm{i}c^{\uparrow\uparrow} & 0 \\ 0 & 1 + a^{\downarrow\downarrow} + \mathrm{i}c^{\downarrow\downarrow} \end{pmatrix} \Psi = \alpha \begin{pmatrix} (1-\alpha) - a^{\uparrow\uparrow} - \mathrm{i}c^{\uparrow\uparrow} & d^{\uparrow\downarrow} - \mathrm{i}b^{\uparrow\downarrow} \\ d^{\downarrow\uparrow} - \mathrm{i}b^{\downarrow\uparrow} & -(1-\alpha) + a^{\downarrow\downarrow} + \mathrm{i}c^{\downarrow\downarrow} \end{pmatrix} \Psi. \tag{24}$$

This equation can be written in the form $H\Psi = E\Psi$ in terms of an effective Hamiltonian

$$H_{ij} = \begin{pmatrix} h_{ij} & \Delta_{ij} \\ \Delta^*_{ji} & -h^*_{ij} \end{pmatrix}, \tag{25}$$

where

$$h_{ij} = \varepsilon_0 \delta_{ij} + \alpha\Delta(a^{\uparrow\uparrow}_{ij} + \mathrm{i}c^{\uparrow\uparrow}_{ij}) \tag{26}$$

$$\Delta_{ij} = \alpha(d^{\uparrow\downarrow}_{ij} - \mathrm{i}b^{\uparrow\downarrow}_{ij}). \tag{27}$$

In writing Supplementary Eq. (24) in the form (25), we have omitted elements $\propto E(a^{\uparrow\uparrow} + \mathrm{i}c^{\uparrow\uparrow})$ since these give insignificant contribution to energies when $|E|/\Delta \ll 1$. The reason is that in the low-energy regime, the expectation value of $(a^{\uparrow\uparrow} + \mathrm{i}c^{\uparrow\uparrow})$ turn out to be of the order of E, and the overall contribution is of the order $\mathcal{O}((E/\Delta)^2)$ as can be verified by perturbation theory. Thus the effective Hamiltonian (25) reduces to the one quoted in the main text and captures the low-energy dynamics accurately to linear order in E/Δ. The accuracy of the model can also be checked by numerically comparing the low-energy spectra of Supplementary Eq. (25) to that obtained without the approximation and we find excellent agreement in the studied regime.

Supplementary Note 2: Thermal Conductance

A quantized thermal conductance is the topological response of the studied system. This observable also provides a powerful diagnostic tool in studying the robustness of the topological properties in the presence of low-energy excitations. Even in gapless systems, the conductance exhibits quantized values as long as the excitations below some finite energy are well-localised compared to the sample size. In the scattering-theory framework, the thermal conductance can be expressed in terms of transmission of quasiparticles. The conductance of the system can be evaluated by connecting it to two semi-infinite leads, each modelled by a simple tight-binding Hamiltonian. The system is then treated as an $L_x \times L_y$ scattering area separating these leads. To make the numerical calculations easier, we fix a number of sites at two opposing edges and only connect these to the leads, while imposing no extra structure on the rest of the scattering area (see Fig.).

The retarded/advanced Green's function of the Shiba glass can be written as

$$G^{-1}_{\mathrm{r,a}}(E) = E - H - \Sigma_{r,a}, \tag{28}$$

where Σ is a self energy term originating from the coupling to the leads. This, in turn, can be found to be

$$\Sigma_{\rm r}(E,m,n)=\begin{cases}\frac{1}{L_y/a+1}\left(\frac{t_{\rm C}}{t_{\rm L}}\right)^2\sum_k\sin(kma)\big(\varepsilon-{\rm i}\sqrt{4t_{\rm L}^2-\varepsilon^2}\big)\sin(kna), & |\varepsilon|<2t_{\rm L}\\ \frac{1}{L_y/a+1}\left(\frac{t_{\rm C}}{t_{\rm L}}\right)^2\sum_k\sin(kma)\big(\varepsilon-{\rm sgn}\,\varepsilon\sqrt{\varepsilon^2-4t_{\rm L}^2}\big)\sin(kna), & |\varepsilon|>2t_{\rm L},\end{cases} \tag{29}$$

where ma, na are the y coordinates of sites connected to the leads, while $t_{\rm C}$ and $t_{\rm L}$ are the hopping parameter between the lead and the Shiba glass, and the hopping parameter within the lead, respectively. Furthermore, we have defined

$$\varepsilon=E-2t_{\rm L}\cos(ka). \tag{30}$$

In the above expressions, k takes values $k(j)=\frac{j\pi}{a(N_y+1)}$ for $j=1\dots N_y$, where N_y denotes the number of fixed sites on each side of the scattering region. The thermal conductance quanta through the scattering area can then be calculated as

$$g={\rm Tr}\left[\Gamma_{\rm L}G_r^{\rm LR}\Gamma_{\rm R}G_a^{\rm RL}\right]. \tag{31}$$

Here, we have defined $\Gamma_{\rm L,R}=-2\,{\rm Im}\,\Sigma_{\rm L,R}$ as well as the matrices $G_{r,a}^{\rm LR}$ and $G_{r,a}^{\rm RL}$. The latter two are the sub-block of the Green's function connecting the leftmost and rightmost edges of the sample – in our setup, the matrix elements connecting the fixed sites on the opposite sides of the Shiba glass.

Supplementary Note 3: Energy vs mobility gap of the Shiba glass in large systems

In the main text, we pointed out that in the infinite-system limit, the Shiba glass is gapless but its topological character as manifested by quantized thermal conductance persists. The purpose of this section is to elucidate on that statement. Finite, reasonably small Shiba glass systems with high adatom density will with high probability be gapped in the bulk when in the topological phase, as can be seen in Supplementary Fig. 2a. However, as the system size increases, the gap will scale down to zero. A system that is spatially infinite but has a finite density of randomly distributed adatoms will with probability 1 contain sparse regions, antipuddles, of any characteristic linear dimension L. For large enough L, this is enough to produce a local topologically trivial region even if the system on average is topological. The boundary of this topologically trivial region will then support some low-but-finite-energy excitations determined by the size of L and vanishing as L grows. Assuming that the moments are distributed independently with a fixed probability per unit area, the probability of getting locally trivial antipuddle configurations is exponentially suppressed as a function of antipuddle size L and generally small for finite systems with densities that put them deep in the topological phase. However, even for finite systems it is possible to create sizable such regions when placing sites randomly on a substrate.

To illustrate the effect of antipuddles, we have considered finite configurations which contain an empty region of specified L and studied the properties of such systems. The results for one such configuration can be seen in Supplementary Fig. 3, where we have plotted its density of states (DOS) and thermal conductance. We have also compared both quantities to the case where the hole is filled.

The low-energy excitations, arising from the antipuddle in the center, are clearly visible in the enhanced DOS around $E=0$. However, the thermal conductance calculated for the configuration clearly exhibits a quantized plateau extending to $|E|=0.3\Delta$, explicitly showing that the low-energy excitations do not destroy the topological response of the system. This implies that the low-energy excitations are localised and the system possesses a well-defined mobility gap between $E=\pm0.3\Delta$ even though the energy gap is negligible. These results illustrate how it is possible that the energy gap scales to zero due to density fluctuations while the mobility gap, which is determined by the average density, will remain finite in the topological phase. Thus, by increasing the system size but keeping the density fixed, we achieve a gapless system with a finite mobility gap and quantized thermal conductance. As long as the density is sufficiently high that the system resides on the topological side of the phase boundary, the states around different antipuddles have negligible overlap and represent localised perturbations that do not destroy the global topology of the system.

Supplementary Note 4: Phase diagrams at different parameters

Metals generally have much clearer separation of Fermi wavelength and superconducting coherence length $k_{\rm F}^{-1}\ll\xi$ compared to the proximity superconducting semiconductors. Also, metals typically have lower spin-orbit splitting at

the Fermi level characterised by parameter $\lambda = \alpha_{\mathrm{R}}/v_{\mathrm{F}}$. To supplement the results presented in the main text, we consider how changing these two parameters affects the topological phase diagram. In general, decreasing λ will have the effect of shifting the topological phase transition to higher densities, whereas increasing ξ will have the opposite effect. These two effects compete and will, in effect, cancel each other out to some extent. In addition, increasing ξ may introduce even new phase transitions, resulting in smaller regions within the overall topological parameter region that may have trivial topology or Chern numbers larger than unity. For sufficiently high densities these generally again vanish, leaving the system with a Chern number of magnitude one. An example of a phase diagram for higher ξ and lower λ can be seen in Supplementary Fig. 2b; note that the system enters the topological phase at lower magnetic moment density (higher $\bar{r}$) than in the ones used in the main text, which is counteracted by the appearance of a new, smaller trivial parameter region within the topological region.

Supplementary References

[1] Pientka, F., Glazman, L. I. & von Oppen, F. Topological superconducting phase in helical Shiba chains. *Phys. Rev. B* **88**, 155420 (2013).

[2] Brydon, P. M. R., Das Sarma, S., Hui, H.-Y. & Sau, J. D. Topological Yu-Shiba-Rusinov chain from spin-orbit coupling. *Phys. Rev. B* **91**, 064505 (2015).

[3] Röntynen J. & Ojanen, T. Topological Superconductivity and High Chern Numbers in 2D Ferromagnetic Shiba Lattices. *Phys. Rev. Lett.* **114**, 236803 (2015).

[4] Röntynen, J. & Ojanen, T. Chern mosaic: Topology of chiral superconductivity on ferromagnetic adatom lattices. *Phys. Rev. B* **93**, 094521 (2016).
